# Generation of High-Resolution Time-Series NDVI Images for Monitoring Heterogeneous Crop Fields

**DOI:** 10.3390/s25165183

**Published:** 2025-08-20

**Authors:** Sun-Hwa Kim, Jeong Eun, Inkwon Baek, Tae-Ho Kim

**Affiliations:** 1 PERPIXEL Inc., Incheon 21984, Republic of Korea; eun2@perpixel.co.kr (J.E.); aaron77@perpixel.co.kr (I.B.); 2 Underwater Survey Technology 21 Inc., Incheon 21999, Republic of Korea; thkim@ust21.co.kr

**Keywords:** spatiotemporal fusion, SSFIT, ESTARFM, PlanetScope, Sentinel-2, NDVI, rice paddy, cabbage fields

## Abstract

Various fusion methods of optical satellite images have been proposed for monitoring heterogeneous farmlands requiring high spatial and temporal resolution. In this study, a three-meter normalized difference vegetation index (NDVI) was generated by applying the spatiotemporal fusion (STF) method to simultaneously generate a full-length normalized difference vegetation index time series (SSFIT) and enhanced spatial and temporal adaptive reflectance fusion method (ESTARFM) to the NDVI of Sentinel-2 (S2) and PlanetScope (PS), using images from 2019 to 2021 of rice paddy and heterogeneous cabbage fields in Korea. Before fusion, S2 was processed with the maximum NDVI composite (MNC) and the spatiotemporal gap-filling technique to minimize cloud effects. The fused NDVI image had a spatial resolution similar to PS, enabling more accurate monitoring of small and heterogeneous fields. In particular, the SSFIT technique showed higher accuracy than ESTARFM, with a root mean square error of less than 0.16 and correlation of more than 0.8 compared to the PS NDVI. Additionally, SSFIT takes four seconds to process data in the field area, while ESTARFM requires a relatively long processing time of five minutes. In some images where ESTARFM was applied, outliers originating from S2 were still present, and heterogeneous NDVI distributions were also observed. This spatiotemporal fusion (STF) technique can be used to produce high-resolution NDVI images for any date during the rainy season required for time-series analysis.

## 1. Introduction

As of 2023, the total agricultural area in Korea was reported to be 1505 thousand hectares, of which rice paddies account for 761 thousand hectares and fields account for 744 thousand hectares [1]. Looking at the growing stages of rice in Korea, the preparation of seedbeds and transplanting of rice seedlings takes place from mid-April to early June, full-scale growth takes place from June to August, and harvesting takes place from mid-September to early October. Rice is an annual crop and takes three to six months from germination to growth, heading, and maturation [2]. In particular, the crops grown in Korean fields are very diverse, the cultivated area for each crop is very small, with less than one hectare per farm household, and various items are grown scattered throughout the country [3]. In order to remotely monitor Korea’s small-scale and diverse farmland, an optical satellite with high spatial and temporal resolution is required. Accordingly, the Compact Advanced Satellite 500-4 (CAS500-4), an agricultural and forestry satellite scheduled to be launched in 2026, is designed to monitor the Korean Peninsula with a spatial resolution of five meters and a three-day cycle.

More accurate monitoring of crops is needed during the summer season when most crops grow rapidly, but this period overlaps with the rainy season, limiting the use of optical satellite images for crop monitoring [4,5,6]. As a solution to this problem, a cloud-free composite algorithm was proposed that collects low- and medium-resolution satellite images over a certain period of time to create a single representative image with minimal cloud influence [7]. Cloud-free composite algorithms can be divided into metho for selecting pixels with minimal cloud influence over a certain period and methods for generating new representative pixel values. However, in the case of crop monitoring, a method for selecting optimal pixels is more useful, because the spectral information of the original image is maintained [6]. In particular, among various optimal pixel selection methods, the MNC algorithm is most widely applied in many optical satellite images, including NOAA-AVHRR and Terra/Aqua MODIS, because the process is simple and the cloud removal effect is significant [7,8,9].

When cloud removal is not complete due to the large amount of cloud cover during the long rainy season, or when monitoring time-series changes in crops, an algorithm for restoring missing data is applied. This type of algorithm is referred to as the gap-filling method, and can be divided into temporal interpolation (or smoothing techniques) and spatial filling [10]. In the case of MODIS, it provides the gap-filled, smoothed (GFS) product that applies these two gap-filling techniques, whereas S2 provides various temporal missing correction techniques through a missing correction program called Decomposition and Analysis of Time Series (DATimeS) software [10,11]. In a previous study, spatial and temporal gap-filling software for NDVI images from S2 satellites was developed for the Korean rice paddy area; this software presented an RMSE of less than 0.15 [12]. Recently, high-resolution satellite constellations such as PS have been utilized for crop monitoring [13,14,15,16], and although they can be taken at short intervals using multiple satellites, there are limitations to this approach, such as the difficulty in generating time-series data due to the cost [17]. In addition, differences occur between satellite constellations, and additional normalization may be required for wide-area monitoring or time-series analysis [18,19].

Satellite sensors with various resolutions are available, and various fusion techniques between multiple satellite images have been proposed to resolve the trade-off between resolutions [20,21]. For example, the STF algorithm was applied to Landsat series and MODIS data to produce high spatial resolution reflectance data for a desired date to monitor crop growth [21,22]. STF algorithms can be broadly divided into pair-based fusion methods such as the spatial and temporal adaptive reflectance fusion method (STARFM) [20], enhanced STARFM (ESTARFM) [23], and regression model fitting, spatial filtering, and residual compression (Fit-FC) [24], and time-series-based fusion methods such as SSFIT [21,22]. The pair-based method is a technique that generates fusion data with dates similar to those of input data based on the statistical relationship between input pairs. It is simple to apply, but its accuracy varies depending on the number or distribution of the input data, and it has the disadvantage of not considering temporal pattern characteristics [21]. On the other hand, SSFIT can generate high-resolution images for a desired date without high-resolution satellite images that are not affected by clouds, and requires a time-series pattern to be input [22]. These fusion methods were developed based on reflectance and have recently been applied to MODIS and Landsat NDVI, suggesting their potential for use [22]. Recently, there has been an increase in the number of cases of fusing PS and S2 for crop monitoring, but most have been applied using spatiospectral fusion methods, and there have been no cases where a fusion method for time-series analysis has been applied [25,26,27].

This study applied the spatiotemporal gap-filling method to S2 images acquired from 2019 to 2021, which showed various precipitation amounts over relatively wide and homogeneous rice fields as well as narrow and heterogeneous cabbage fields, to generate S2 NDVI with fewer missing values and improved temporal consistency. Two representative fusion methods (SSFIT and ESTARFM) were then applied to these data and to PS data, respectively. Through this process, we produced three-meter NDVI for a desired date and compared the fusion results of the two methods to suggest a fusion method suitable for small and heterogeneous farmland. The specific research objectives are as follows:

– To present a fusion technique capable of providing NDVI with a high spatial resolution of 3 m, enabling effective monitoring of vegetation indices in small, heterogeneous fields.
– To compare the representative fusion techniques, ESTARFM and SSFIT, and to determine the optimal method for fusing S2 data, which provides time-series information, with PS data, which offers high spatial resolution.
– To evaluate the performance of the fusion techniques during periods of high cloud cover.

## 2. Materials and Methods

### 2.1. Study Area and Data Used

**Figure 1:**
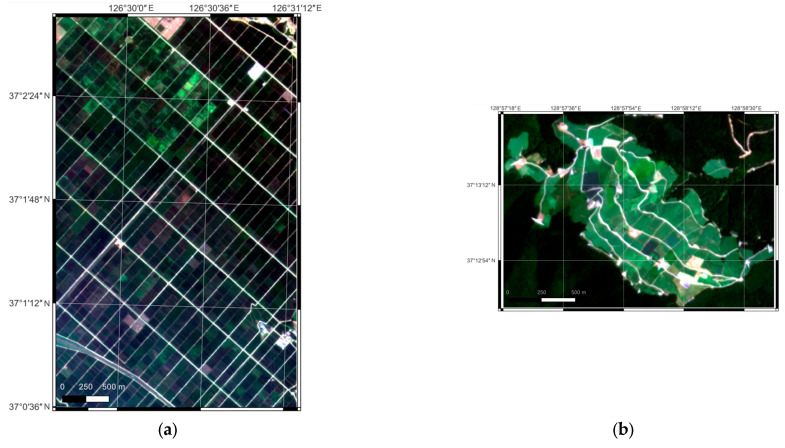
PS natural color composite images of the two study areas: (**a**) Dangjin rice paddy; (**b**) Taebaek cabbage fields.

The study area selected was the main agricultural land in Korea, which comprises rice paddies and dry fields. In this area, the type of crop often changes every year, so a highland cabbage field that grows the same crop every year was selected for time-series analysis. The rice paddy area selected was Waemok Village, Dangjin, Chungcheong Province (Figure 1a). Rice planting began in May and harvesting began in October. As shown in Figure 1, the rice paddy area shows a relatively homogeneous distribution compared to the fields. Another study area selected was a highland cabbage field in Maebongsan, Taebaek, Gangwon-do (Figure 1b). Because it is a high-altitude area, only cabbage is cultivated here; the planting and cultivation periods differ depending on the owner of each plot. Cabbage is planted from May to June and harvested between August and September. Recently, the region has been suffering from soft rot due to the effects of global warming, which has led to a decrease in harvest yield.

Sentinel-2 (S2), a medium-resolution satellite with high periodic resolution, and PlanetScope (PS), a high-resolution satellite, were used to collect images in the two study areas. S2 Level 2A images provide a spatial resolution of 10 m, and are taken by two satellites intersecting each other, providing images of the same area every five days. This is an atmospheric-corrected L2A reflectance image using Sen2Cor software, provided by the Copernicus Open Access Hub (https://dataspace.copernicus.eu (accessed on 29 October 2021)). The red band (650–680 nm) and near-infrared band (785–899 nm) of S2 are used to derive time-series NDVI patterns. Another data to be used for fusion is PS L3B, a satellite constellation image data that provides a spatial resolution of 3 m and can be taken on any desired date, but requires a fee. PS reflectance data is data that has undergone 6SV-based atmospheric correction. The NDVI was created using the reflectance of the red band (650–680 nm) and the near-infrared band (845–885 nm). PS NDVI images are divided into training data input to the STF model and test data to verify the accuracy of the fusion result.

The target period selected was from May to October to capture the growing cycle of the rice paddy, and from May to September for the fields. In addition, data from 2019 to 2021 showing average precipitation and data from 2020 showing about 200 mm higher precipitation than the average from July to October were used for time-series analysis [5], as shown in Table 1.

### 2.2. Methods

**Figure 2:**
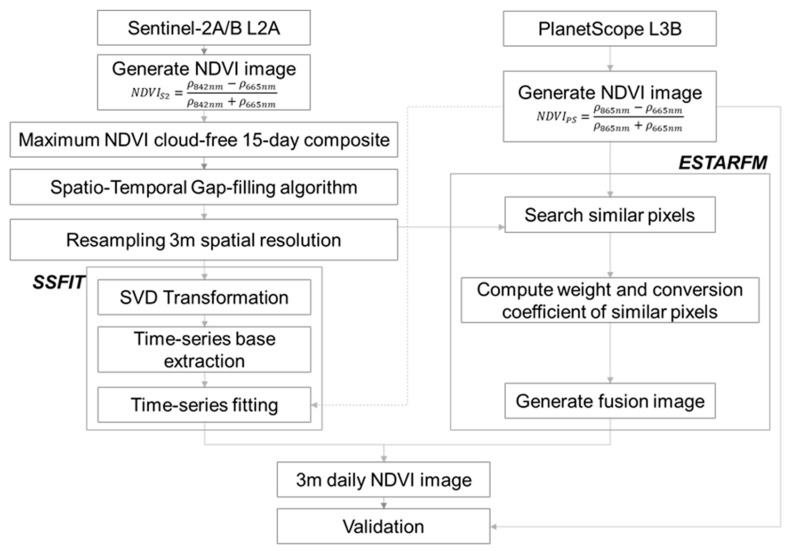
Flowchart for fusion processing of S2 and PS NDVI images.

In order to fuse S2 time series NDVI data with PS data providing 3 m NDVI, this study applied representative two STF methods, SSFIT and ESTARFM, respectively. As shown in Figure 2, preprocessing is performed to input each STF method. Verification was performed using the data fused by the two methods and the PS test data. The following contents describe in detail the preprocessing process (2.2.1) and two STF methods (2.2.2 and 2.2.3) of the two satellite images, and the verification method (2.2.4) of the two results.

#### 2.2.1. Preprocessing

After generating NDVI using the red and near-infrared bands of the S2 L2A data, data is acquired every 15 days, and a MNC process was performed to create a representative image with pixels showing the maximum NDVI per pixel to minimize the influence of cloud cover. To minimize missing data due to remaining cloud areas, a spatial gap-filling process was performed to replace missing pixels with the average NDVI of nearby pixels in small missing areas of 5 pixels or less. Next, Gaussian process regression (GPR), the most commonly used technique for temporal gap-filling, was performed, which requires two images taken before the missing date and one image taken after it [11]. This spatiotemporal gap-filling technique has been modularized and used in Python 3.8.15, showing an RMSE of 0.173 and taking 5 min of processing time per scene [12]. Through this process, S2 time series NDVI data from 2019 to 2021 with minimal cloud impact were generated, and S2 data to be used for two STF methods were prepared. The S2 time-series NDVI data from 2019 to 2021, when the cloud impact was minimized, were resampled to 3 m to match the PS data and inputted into the two STF models. The PS2 L3B data was also used to generate an NDVI image using two bands, and was divided into training data, which was used as input data for the two fusion models, and test data, used to verify the fusion methods. The following Section 2.2.2 and Section 2.2.3 describe the two types of STF.

#### 2.2.2. SSFIT Method

The SSFIT is a fusion algorithm developed based on time-series MODIS and Landsat EVI data, and presents relatively higher accuracy than other STF methods for crop monitoring [21,22]. First, SVD (singular value decomposition) transformation is applied to derive base (*b_i_*), representing the pattern of time-series NDVI from time-series S2 data. Through SVD transformation, an orthogonal matrix providing spatial information of S2, a diagonal matrix composed of singular values, and bases, which are right-singular row vectors providing time series information, are derived. In this study, *b_i_* was derived by year. Next, by inputting the PS NDVI (*f*) and the base (*b_i_*) of S2 into Equation (1), base coefficients (*a_i_*) were calculated using the least squares method.

(1) $$f\left(x,y,t\right)=\sum_{i=1}^{n_{b}}a_{i}\left(x,y\right)\times b_{i}\left(x,y,t\right)+\varepsilon (x,y,t)$$

where *f*(*x*, *y*, *t*) is the PS NDVI value of the pixel located at (*x*, *y*) observed on date *t*, *b_i_*(*x*, *y*, *t*) is the *i*th base obtained from S2, *a_i_*(*x*, *y*) is base coefficients of the *i*th base, *n_b_* is the number of bases, and *ε* is the model residual [22].

The coefficients are calculated for each pixel, and the 3 m NDVI image for a desired date are generated using these coefficients, which are implemented in Python.

#### 2.2.3. ESTARFM Method

ESTARFM is an algorithm that combines spectral mixing analysis with STARFM, a representative weight-based fusion algorithm, to achieve higher accuracy in heterogeneous regions [23,28]. A search window is set to search for spatially adjacent neighboring pixels at the predicted location, and the spatial, spectral, and temporal similarities between the input S2 and PS pair data and the PS data of the predicted date are calculated as weights. When the search window size is *w*, the 3 m high-resolution NDVI value at the center pixel (*x_w/2_*, *y_w/2_*) of the search window on the prediction date *t_p_* is predicted as the sum of the weighted input data, as in Equation (2).

(2) $$f\left(x_{w/2},\;y_{w/2},\;t_{p}\right)=\sum_{k=1}^{K}\left\{T_{k}\times \left[f(x_{w/2},\;y_{w/2},\;t_{0})\right]+\sum_{i=1}^{w}\sum_{j=1}^{w}W_{ij}\times v\left(x_{i},y_{j}\right)\times \left[c\left(x_{i},y_{j},t_{p}\right)-c(x_{i},y_{j},t_{0})\right]\right\}$$

where *k* is the number of pairs of S2 and PS data acquired on the same date *t_k_*, *f* is the S2 NDVI, *c* is the PS NDVI image, and *T_k_* is the temporal weight for the intensity of change in the time-series NDVI. *W* is the weight for spatial and spectral information, and a high weight is assigned to data that exhibits similar spectral characteristics and are spatially close within the search window. The coefficient *v* that converts S2 to PS, and considers the difference in spatial resolution. In this study, the Python implementation code of ESTARFM provided on the webpage (https://xiaolinzhu.weebly.com/open-source-code.html, accessed on 29 April 2025) was used.

#### 2.2.4. Validation Method

In this study, two STF fusion results corresponding to the dates of independent PS test data were predicted and compared with the predicted data and the PS NDVI test data. To verify whether the actual high-resolution NDVI was generated, a qualitative comparison is performed visually on 10 m S2, 3 m PS, and fused 3 m NDVIs. In addition, for quantitative analysis, correlation and RMSE values were calculated based on the PS test data, and time-series analysis of S2 time-series NDVI, PS, and the fused NDVI image was performed. In these qualitative and quantitative validations, this study employed various comparative perspectives. First, we analyzed which STF method is more efficient in heterogeneous areas by comparing the two representative STF results for homogenous rice paddy area and heterogeneous cabbage fields. In addition, we compared the STF results between 2019 and 2021, which showed normal precipitation, and for 2020, which showed high precipitation (high cloud cover).

## 3. Results

### 3.1. Qualitative Evaluation of Time-Series Fused NDVI Data

**Figure 3:**
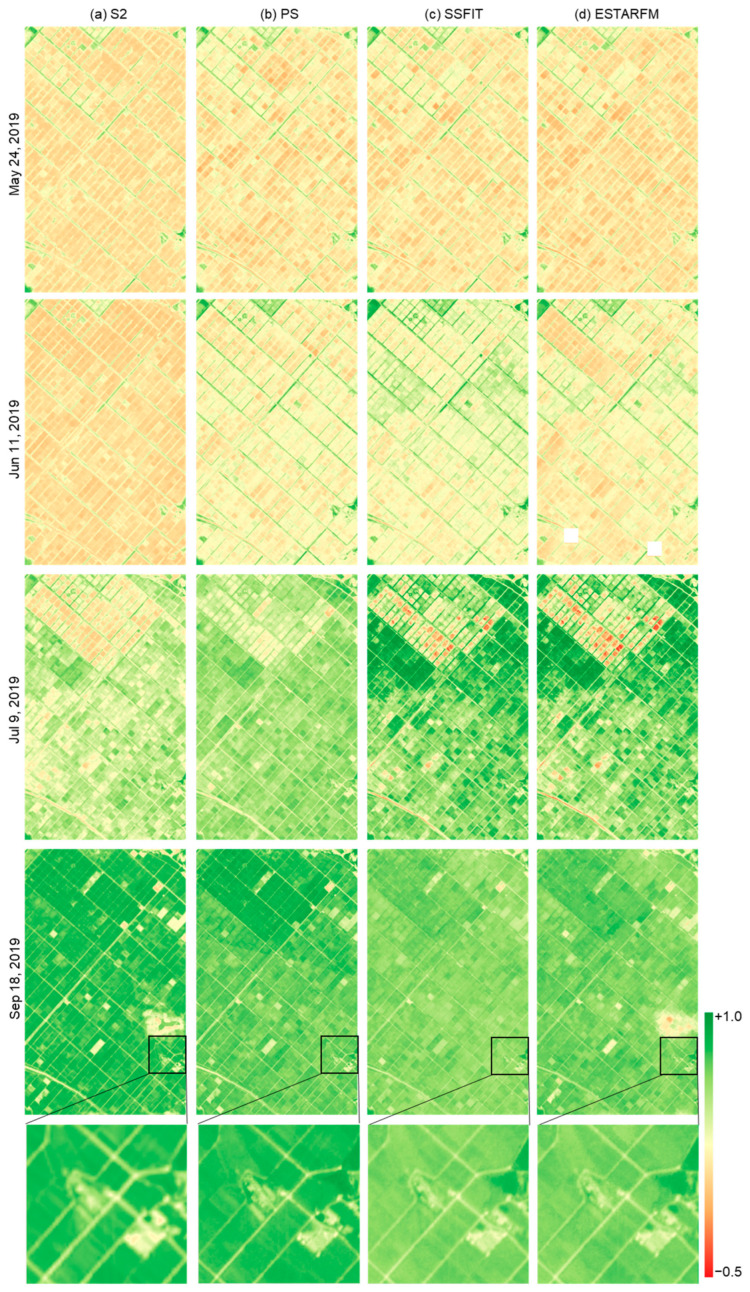
NDVI images of the rice paddy area on the prediction dates in 2019.

**Figure 4:**
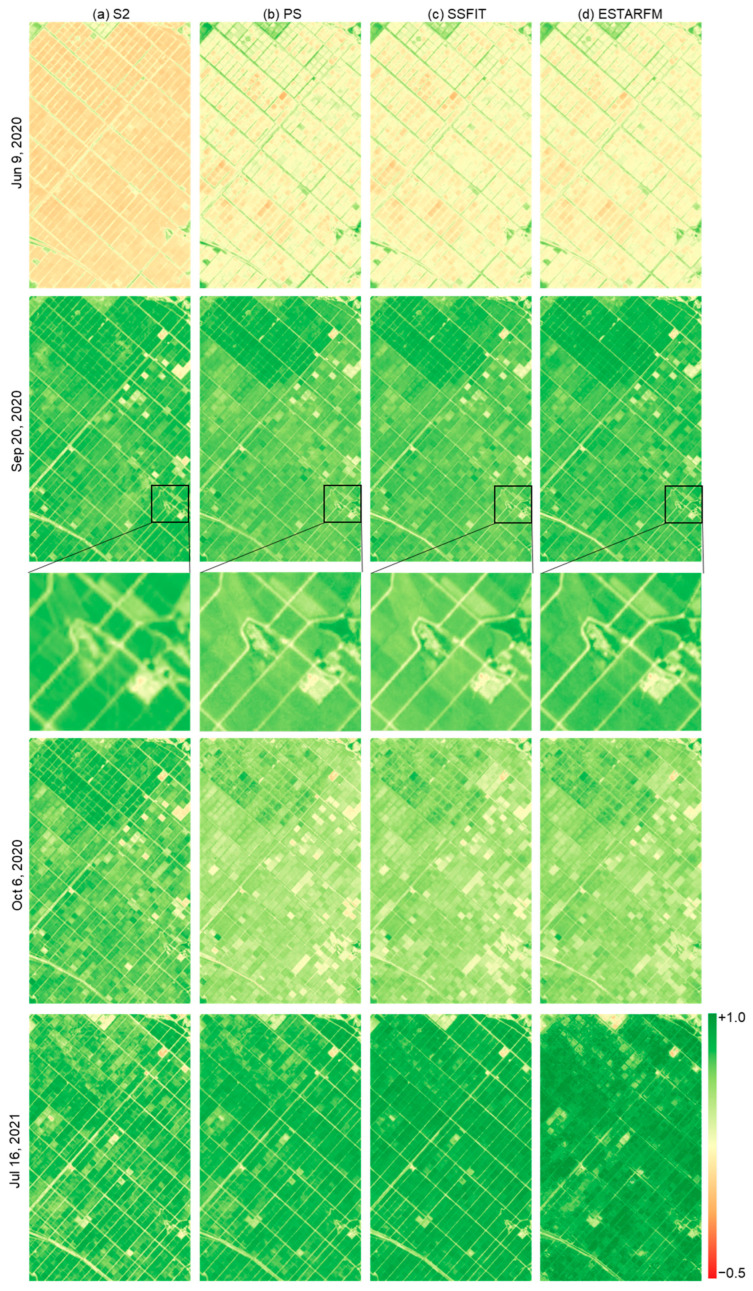
NDVI images of the rice paddy area on the prediction dates from 2020 to 2021.

**Figure 5:**
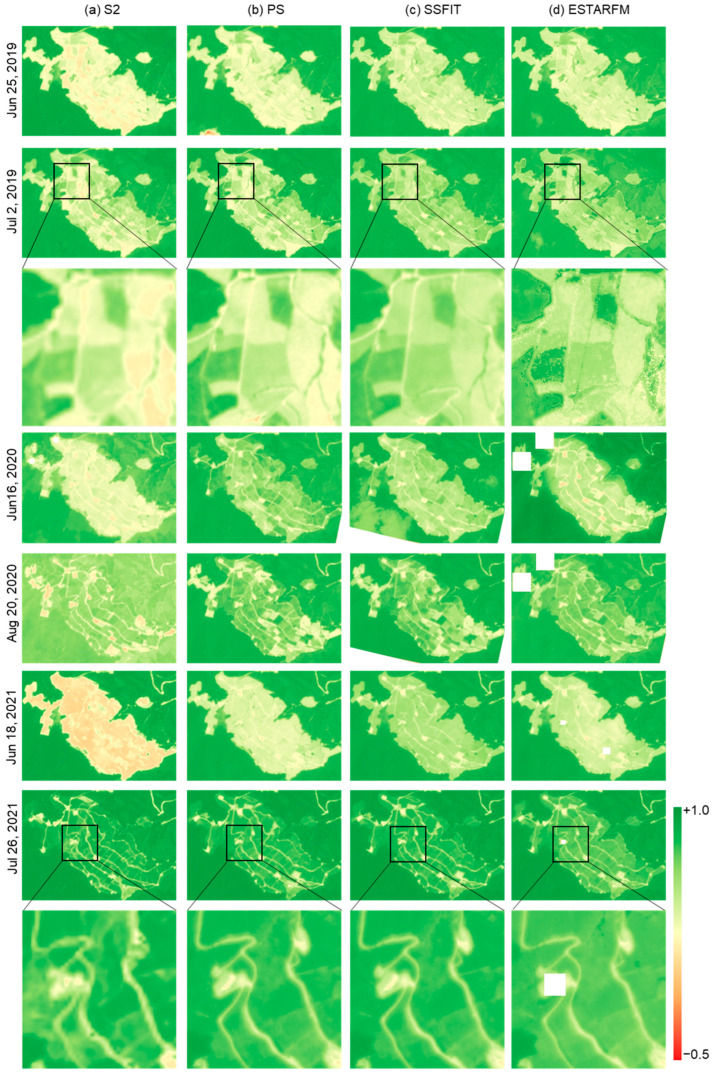
NDVI images of the cabbage field area on the prediction dates from 2019 to 2021.

**Figure 6:**
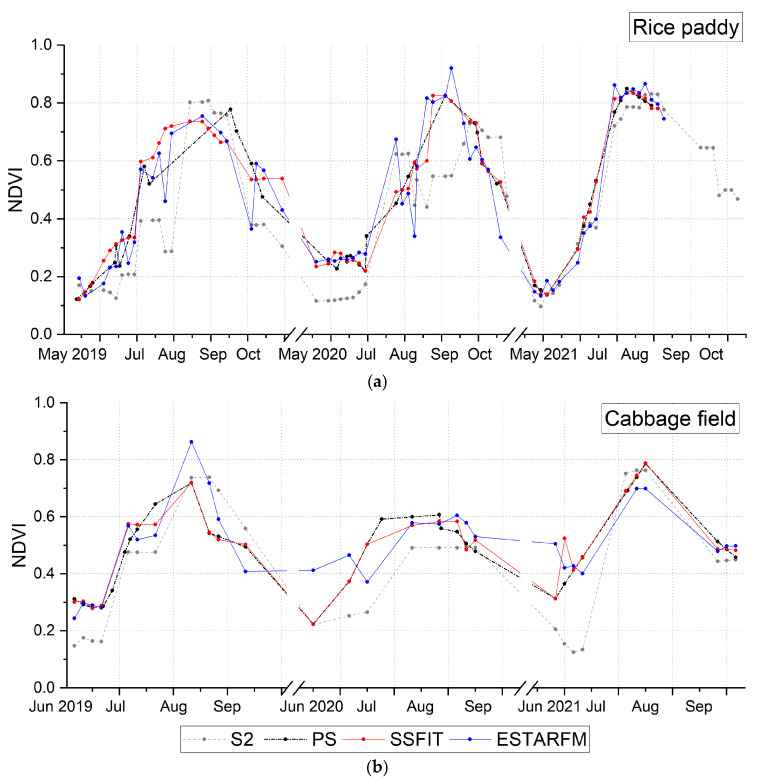
Observed and predicted time-series NDVI patterns from 2019 to 2021: (**a**) rice paddy; (**b**) cabbage field.

To evaluate the qualitative quality of the NDVI images synthesized by the two techniques, the S2, PS, and fused NDVI images were visually compared for rice fields and cabbage fields (Figure 3, Figure 4 and Figure 5). The S2 NDVI image is a composite of data from a 15-day period and is different from the PS image taken on that date. Looking at the NDVI of the rice paddy area in 2019 (Figure 3), May and June showed very low NDVI due to the rice planting season, then increased until September and decreased after harvest in October. In addition, differences in harvest times by plot were observed. In the highland cabbage fields, the NDVI increases significantly between June and July, and decreases from mid-August when harvest is made. The NDVI difference in cabbage fields by year was large compared to that in the rice paddy area. The PS and fusion results on both sites mostly showed similar NDVI distributions, but there were differences in the NDVI between the images of the paddy field area on 9 July 2019 and 18 September 2019 and the images of the cabbage field area on 16 June 2020 and 18 June 2021. Most of these data were acquired during a period when the rate of vegetation change was rapid. Looking at the time-series NDVI pattern (Figure 6), this difference is caused by a large difference in NDVI of the test data taken at a similar time (<10 days) as the PS training data used for prediction. In addition, for the cabbage fields in 2020 and 2021, where the number of PS training images was small, the differences between the fused and the PS test NDVI images increased (Figure 5 and Figure 6).

In addition, when examining the partial images (Figure 3, Figure 4 and Figure 5), improved spatial resolution was confirmed in the 3 m fused NDVI image compared to the 10 m S2 NDVI image, and more detailed boundaries were shown, especially in the cabbage field. Comparing the results of the two fusion methods, we can see that the influence of the remaining clouds in S2 on 18 September 2019 was also observed in ESTARFM. In addition, ESTARFM showed areas that were not predicted or had an inhomogeneous NDVI distribution compared to SSFIT results on specific dates. This is a phenomenon that occurs because ESTARFM estimates only used NDVI near the forecast date, unlike SSFIT, which utilizes time-series pattern information, and thus the quality information of the data used is directly reflected.

Figure 6 shows the time-series NDVI values for S2 and PS data from 2019 to 2021 for rice paddies and cabbage fields, along with a composite NDVI generated on the S2 acquisition date. The fusion method utilizes only the time-series features of the S2 data and the spatial information of the PS data, resulting in a fusion result similar to the PS NDVI. The PS data were captured mostly on clear days, so on days when the PS was not observed due to cloudy weather, the fusion results provide NDVI values. Accordingly, the fusion results show a more stable time-series pattern than the original PS data. In contrast, the NDVI data of S2 shows significantly greater temporal variation, with significant fluctuations in NDVI in 2019 and 2020, except in 2021, when cloud cover was minimal. Compared to the PS data, which was captured only on clear days, the S2 data are significantly affected by cloud cover. In particular, the cabbage field shows a very low NDVI before seeding. At this time, the field is bare land and shows an NDVI lower than the cloud, which is an abnormal phenomenon that occurs due to the phenomenon of pixels affected by clouds being selected during period composition. On the other hand, compared to the PS taken on a cloudless day, the S2 NDVI shows a low NDVI throughout the field, as shown in Figure 5a. The fusion technique shows a time-series pattern that resolves the limitations of both PS and S2 data, and in particular, the SSFIT method presents a more stable time-series pattern than ESTARFM.

### 3.2. Quantitative Evaluation of Two STF Results Using PS Test Images

**Figure 7:**
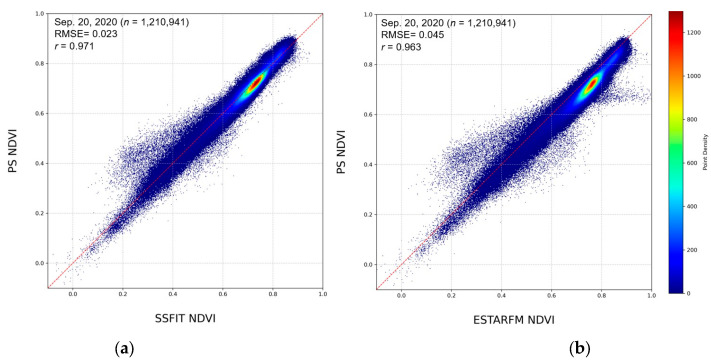
Scatter plots of the STF NDVI and the PS test NDVI for the rice paddy on 20 September 2020: (**a**) SSFIT; (**b**) ESTARFM.

To quantitatively evaluate the fused NDVI image, the one-to-one correspondence distribution, correlation, and RMSE with the PS test NDVI image were calculated. As shown in Table 2 and Table 3, the STF results in the rice paddy area showed an RMSE of 0.02 to 0.16 and a correlation of 0.80 or higher based on the PS test NDVI image, while in the cabbage fields, the RMSE was lower than 0.1 and a high correlation of 0.87 or higher was observed. Although accuracy differences are small, the scatter plot shows that SSFIT is distributed closer to a one-to-one line than ESTARFM, is more coherent, and has a lower RMSE (Figure 7).

For SSFIT, it took four seconds to process based on NDVI images in the rice paddy, whereas ESTARFM took five minutes to process. The tested PC specifications are AMD Ryzen 9 5900X 12-Core Processor 3.70 GHz, RAM DDR5 PC5-44800 128GB, and GPU NVIDIA GeForce RTX 3060 Ti. The ESTARFM code took a longer amount of processing time because it utilizes a process of calculating similarity between input images through a search window.

## 4. Discussion

### 4.1. Applicability of SSFIT and ESTARFM Techniques

Most of the existing studies on the fusion of S2 and PS have used the spatial-spectral fusion (SSF) technique [25,26,27]. With the construction of S2 time-series data, the application of the spatiotemporal fusion (STF) technique has become feasible. The STF technique has been predominantly applied to fuse Landsat and MODIS time-series data [20,21,22,23], and performance comparison studies among various fusion techniques have also been conducted [21,22]. This study is the first to apply and compare two representative STF techniques that demonstrate high accuracy with time-series S2 and PS data. In particular, our goal was to identify the optimal fusion method for the purpose of heterogeneous farmland monitoring.

SSFIT requires a time-series of S2 data and two or more pairs of S2 and PS data, enabling the estimation of daily NDVI over the S2 time span after model construction. ESTARFM requires two pairs of S2 and PS data, as well as S2 data for the forecast date. Since S2 data are acquired in a 5-day cycle, estimating daily NDVI is not feasible with this method. Regarding processing methods, SSFIT can quickly estimate NDVI for a desired date by simply constructing the S2 time series, but it is limited by the need to develop the time-series data. In contrast, ESTARFM is a straightforward method if data from dates close to the target date are available. However, it often underestimates NDVI values compared to the original PS data, and the quality of the estimation is highly affected by the limited number of input data points. The influence of the quantity and temporal distribution of input data on the accuracy of fusion techniques has been discussed in previous studies [21,23]. In this study, we attempted STF with S2 by utilizing a greater number of PS data points than in previous research, resulting in effective fusion outcomes. Nonetheless, practical limitations such as cost raise questions about the applicability of the STF technique when fewer PS data are available. In theory, using two or more PS images can enable prediction, but the accuracy may decline. Additionally, if a large number of PS images are available, they can be directly used for time-series monitoring without fusion. However, as shown in the NDVI time-series pattern, PS NDVI exhibits significant variability over days, complicating the analysis of stable NDVI time series compared to S2 NDVI. Therefore, predicting NDVI on a specific date with an STF method like SSFIT is considered more useful. Furthermore, in 2019, which had fewer data, the accuracy was slightly lower than in 2020, which had a larger dataset, while 2021—similar in data volume to 2019 but with data concentrated until August—showed relatively higher accuracy than 2019. This indicates that both the quantity and temporal distribution of data significantly influence the accuracy of STF predictions. Additional analysis is required to quantify how the distribution and number of training data affect STF performance.

Since predicting and analyzing time-series NDVI can be challenging for real-time agricultural monitoring, the high-quality, daily NDVI data produced by SSFIT can serve as valuable input for yield prediction and crop biophysical modeling. This provides wide-area, long-term data provided solely by satellite sensors, enabling rapid NDVI image generation for specific regions and dates using AI techniques.

### 4.2. Comparison of Fusion Results in Rice Paddies and Fields

Previous studies have shown that the heterogeneity of the target area greatly influences fusion outputs [21,22,23,25]. In this study, we selected homogeneous rice paddies and heterogeneous fields as the study areas to analyze how land cover composition affects fusion results. Compared to rice paddies, cabbage fields typically have shorter growth cycles and narrower NDVI ranges. Additionally, rice paddies tend to exhibit similar NDVI patterns throughout the year, whereas cabbage fields display more distinct NDVI fluctuations. The high spatial resolution of 3 m achieved through fusion is thus more valuable for monitoring heterogeneous fields like cabbage than for homogeneous rice paddies.

As illustrated in Figure 5, the heterogeneous distribution of NDVI variation in small cabbage fields is clearly visible in the fusion results. Moreover, the RMSE values calculated against PS data show that prediction accuracy in field areas is comparable to that of paddies (Table 2 and Table 3). However, as shown in Figure 6b, ESTARFM often deviated more from PS data than SSFIT, and it tended to produce excessively high NDVI values before sowing and post-harvest. This discrepancy is likely due to the quality of the input data used in ESTARFM. Since cabbage fields grow faster than rice paddies, SSFIT is preferable for generating high-resolution NDVI images with a rapid update cycle to support effective analysis.

### 4.3. Influence of Clouds on Fusion Results

Before performing fusion, the S2 data were processed with cloud-free compositing and spatiotemporal gap-filling techniques to obtain more stable time-series information. However, during certain years and especially in the rainy season with heavy cloud cover, these methods could not completely eliminate cloud effects, leaving residual noise in the data. As in previous studies, this study selected high-resolution satellite images with minimal cloud impact [22].

In this study, to evaluate the performance of the fusion technique during periods of high cloud cover, we analyzed data from 2020, a year with above-average cloud coverage (2019 and 2021 had even higher cloud cover). As shown in Figure 6, the S2 data exhibited low and highly variable NDVI values in 2021 due to extensive cloud cover. Meanwhile, the PS data, which were collected only on relatively clear days, along with the NDVI predicted through fusion, showed their highest values in August and September. In 2020, heavy rainfall contributed to overall lower NDVI values in the fields compared to other years. Even during the rainy months of July and August in 2019 and 2021, the NDVI data predicted using the SSFIT method showed more stable and higher NDVI values than the low-variability S2 data. In the rice paddies of 2021, the S2 data displayed more consistent NDVI, partly due to less cloud interference. The composite data closely matched the S2 and PS data, displaying typical rice NDVI patterns.

This study compared the accuracy of two STF techniques across different land covers and varying cloud conditions. However, as emphasized in previous research [21], further analysis is necessary to understand how the number, distribution, and specific configuration (e.g., window size) of input data influence the effectiveness of STF algorithms based on the intended application.

## 5. Conclusions

Two representative STF methods, SSFIT and ESTARFM, were applied to S2 and PS NDVI to produce 3 m NDVI images for the desired date. The results of applying both techniques showed similar spatial resolution and NDVI distribution to PS data. The fused data provided more detailed NDVI information in the heterogeneous field with improved spatial resolution. Compared with the ESTARFM method, the SSFIT method provided more accurate and homogeneous NDVI information by reflecting the time-series NDVI information throughout the year, which was less affected by errors or inaccuracies in the input data. In addition, SSFIT’s processing speed is significantly faster, so it can be used more efficiently. While it is difficult to obtain clear images during the rainy season with S2 data acquired periodically, the NDVI of a desired day can be estimated through fusion with PS images acquired on clear days, making time-series NDVI analysis easier. In the future, quantitative analysis will be conducted on how the number and distribution of input data affect the STF results.

## Figures and Tables

**Table 1:** Specifications of two study areas and dataset used.

Site	Rice Paddy	Highland Cabbage Field
Location (Area)	37.03071° N, 126.50696° E (1042.5 ha)	37.21837° N, 128.96659° E (102.6 ha)
Growing period of crops	The fields are filled with water in April, rice seedlings are planted in May, and harvested in October	Sowing takes place from May to June, and harvesting takes place from August to September. The sowing period, growing status, and harvesting period vary depending on the plot
S2 L2A (10 m, 5 days)	May 13, 2019~October 25, 2019 (24 scenes) May 12, 2020~October 24, 2020 (26 scenes) May 12, 2021~October 24 2021 (25 scenes)	Jun 4, 2019~September 7, 2019 (11 scenes) Jun 8, 2020~September 6, 2020 (8 scenes) Jun 13, 2021~September 21, 2021 (10 scenes)
PS Dove L3B (3 m, occasional)	May 11, 2019~October 9, 2019 (13 scenes) April 29, 2020~October 6, 2020 (21 scenes) May 12, 2021~August 15, 2021 (14 scenes)	Jun 4, 2019~September 7, 2019 (14 scenes) Jun 8, 2020~September 6, 2020 (10 scenes) Jun 13, 2021~September 21, 2021 (9 scenes)

**Table 2:** Accuracy of the STF NDVI image, estimated based on the PS test NDVI image of the rice paddy.

Date		SSFIT		ESTARFM	
		RMSE	Correlation	RMSE	Correlation
2019	May 24 Jun 11 Jul 9 Sep 13 Sep 18	0.068 0.096 0.151 0.139 0.110	0.860 0.840 0.851 0.828 0.801	0.073 0.078 0.158 0.140 0.102	0.849 0.837 0.833 0.793 0.693
2020	Jun 9 Jun 22 Sep 20 Oct 6	0.024 0.125 0.023 0.046	0.994 0.893 0.971 0.914	0.029 0.107 0.045 0.062	0.979 0.909 0.963 0.932
2021	Jul 16	0.057	0.951	0.131	0.723

**Table 3:** Accuracy of the STF NDVI image, estimated based on the PS test NDVI image of the cabbage fields.

Date		SSFIT		ESTARFM	
		RMSE	Correlation	RMSE	Correlation
2019	Jun 25 Jul 2	0.073 0.056	0.969 0.988	0.069 0.089	0.966 0.944
2020	Jun 16 Aug 20	0.084 0.049	0.883 0.971	0.134 0.064	0.874 0.943
2021	Jun 18 Jul 28	0.104 0.004	0.980 0.999	0.048 0.032	0.989 0.984

## Data Availability

The data presented in this study are available on request from the corresponding author.

## Abbreviations

The following abbreviations are used in this manuscript:

NDVI	Normalized Difference Vegetation Index
STF	Spatiotemporal Fusion
SSFIT	Spatiotemporal fusion method to Simultaneously generate Full-length normalized difference vegetation Index Time series
ESTARFM	Enhanced Spatial and Temporal Adaptive Reflectance Fusion Method
S2	Sentinel-2A/B
PS	PlanetScope
MNC	Maximum NDVI Composite
RMSE	Root Mean Square Error
